# A single‐chain antibody construct with specificity of a natural IgM antibody reduces hepatic ischemia reperfusion injury in mice

**DOI:** 10.1111/jcmm.18291

**Published:** 2024-04-10

**Authors:** Zhi Yang, Chunmei Li, Yongqin Wang, Wei Dong, Moujie Yang, Junfei Jin

**Affiliations:** ^1^ Guangxi Key Laboratory of Molecular Medicine in Liver Injury and Repair the Affiliated Hospital of Guilin Medical University Guilin Guangxi China; ^2^ Guangxi Health Commission Key Laboratory of Basic Research in Sphingolipid Metabolism Related Diseases the Affiliated Hospital of Guilin Medical University Guilin Guangxi China; ^3^ China–USA Lipids in Health and Disease Research Center Guilin Medical University Guilin Guangxi China; ^4^ Laboratory of Hepatobiliary and Pancreatic Surgery the Affiliated Hospital of Guilin Medical University Guilin Guangxi China

**Keywords:** C2 scFv, complement, IgM antibodies, IRI

## Abstract

Natural immunoglobulin M (IgM) antibodies have been shown to recognize post‐ischemic neoepitopes following reperfusion of tissues and to activate complement. Specifically, IgM antibodies and complement have been shown to drive hepatic ischemia reperfusion injury (IRI). Herein, we investigate the therapeutic effect of C2 scFv (single‐chain antibody construct with specificity of a natural IgM antibody) on hepatic IRI in C57BL/6 mice. Compared with PBS‐treated mice, C2 scFv‐treated mice displayed almost no necrotic areas, significant reduction in serum ALT, AST and LDH levels, and significantly reduced in the number of TUNEL positive cells. Moreover, C2 scFv‐treated mice exhibited a notable reduction in inflammatory cells after hepatic IRI than PBS‐treated mice. The serum IL‐6, IL‐1β, TNF‐α and MPC‐1 levels were also severely suppressed by C2 scFv. Interestingly, C2 scFv reconstituted hepatic inflammation and IRI in Rag1^−/−^ mice. We found that C2 scFv promoted hepatic cell death and increased inflammatory cytokines and infiltration of inflammatory cells after hepatic IRI in Rag1^−/−^ mice. In addition, IgM and complement 3d (C3d) were deposited in WT mice and in Rag1^−/−^ mice reconstituted with C2 scFv, indicating that C2 scFv can affect IgM binding and complement activation and reconstitute hepatic IRI. C3d expression was significantly lower in C57BL/6 mice treated with C2 scFv compared to PBS, indicating that excessive exogenous C2 scFv inhibited complement activation. These data suggest that C2 scFv alleviates hepatic IRI by blocking complement activation, and treatment with C2 scFv may be a promising therapy for hepatic IRI.

## 1 INTRODUCTION

Hepatic ischemia reperfusion (IR) is a major cause of liver damage, organ rejection and sometimes hepatic failure, and is commonly happened following hepatic lobectomy and hepatic transplantation.^1^, ^2^, ^3^, ^4^ IR injury (IRI) in liver is an inevitable outcome of liver transplantation and resection surgery, so understanding the pathogenesis of liver IRI is important for optimizing surgery success.^5^, ^6^ During hepatic IR, sudden intense oxidative stress and an inflammatory response can directly lead to hepatocyte injury and necrosis.^7^, ^8^, ^9^, ^10^, ^11^ Inflammatory cytokines and chemokines produced by damaged hepatocytes are involved in the recruitment of immune cells to the liver, causing immune activation together with activation of the complement cascade, culminating in hepatic IRI.^3^, ^12^, ^13^, ^14^, ^15^ Interestingly, complement activation products also participate in the clearing of dead hepatocytes after liver IR, and promote hepatocyte proliferation and initiation of liver repair processes.^16^, ^17^ A natural IgM antibody is an essential component of the immune system and is also a key element in the complement activation pathway.

Studies have found that IgM can activate complement and drive IRI in various organs and tissues.^18^, ^19^, ^20^, ^21^, ^22^, ^23^, ^24^ A specific IgM mAb, B4, was found to induce intestinal IRI in Rag1^−/−^ mice in previous studies.^19^, ^20^ When administered to Rag1^−/−^ mice, B4 mAb activates complement and recruits neutrophils, thereby reconstituting intestinal IRI in otherwise protected Rag1−/− mice.^20^ Additional studies have shown that the B4 epitope is expressed following spinal cord injury,^22^ ischemic stroke^21^ and cardiac transplant^23^ and the B4 epitope thus represents a potential therapeutic target. The models mentioned above showed that B4‐Crry, a combined protein made up of B4 scFv connected to Crry complement inhibitor, hindered IgM attachment and reduced IRI. With regard to the C2 IgM mAb, a study showed that C2 mAb enhanced arthritis related injury and that C2‐Crry reduced inflammation and arthritis in a murine model.^24^ These results suggest that injury‐specific neoepitopes recognized by IgM may provide therapeutic targets for IRI.

Notably, Marshall et al.^25^ demonstrated that both B4 and C2 IgM mAbs induced hepatic IRI in Rag1^−/−^ mice that were otherwise protected. Rag1^−/−^ mice are commonly used as animal models to assess the effectiveness of complement inhibitors. Furthermore, B4 scFv reduced hepatic IRI in C57BL/6 mice by blocking IgM binding.^25^ However, how C2 scFv may be involved the pathogenesis of hepatic IRI remains unexplored. Herein, we identified a protective role of C2 scFv against murine hepatic IRI as a result of blocking complement activation, data suggested that C2 scFv could be a promising treatmen for preventing hepatic IRI.

## 2 MATERIALS AND METHODS

### 2.1 Mice

Male C57BL/6 mice were acquired from Hunan SJA Laboratory Animal Co., Ltd. Male Rag1^−/−^ mice were purchased from GemPharmatech LLC. Mice used in the experiments were approximately 8 weeks of age. The mice were quarantined for at least 1 week before the experiments. The Animal Ethics Committee in Guilin Medical University reviewed and approved all animal handling and procedures.

### 2.2 Hepatic IR injury mouse model

First, anaesthetised mice had their liver exposed through midline laparotomy. The portal vein and hepatic artery, which supply blood to the liver, were bluntly isolated and quickly closed with non‐invasive microvascular clamp. Observable colour change from red to pale or earthy grey confirmed successful obstruction of liver blood flow. Sham mice, as control, underwent the same procedure without blood vessel occlusion. After 30 min of ischemia, forceps were removed to allow a 6‐h reperfusion period. Liver and serum samples were then collected for subsequent analysis at the conclusion of the surgical procedure.

### 2.3 C2 scFv purification and treatment

Plasmid pEE12.4‐C2 scFv was kindly provided by Professor Stephen Tomlinson from the Medical University of South Carolina, USA. The Expi293™ Expression System (Gibco, A14635) was used for C2 scFv expression according to the manufacturer's guidelines. Protein extraction and purification were conducted utilizing magnetic beads His‐tag protein purification kit (IDA‐Ni) following the manufacturer's protocol. The obtained proteins were collected and stored at −20°C until use. The safety of C2 scFv treatment were assessed by histopathology and biochemical factor analysis (Figure S1).

Mice underwent a period of 30 min of ischemia followed by 6 h of reperfusion. In therapeutic investigations, C57BL/6 mice were injected intraperitoneally with C2 scFv (20 μg) 5 min prior to reperfusion. For reconstitution experiments, Rag1^−/−^ mice were administered intraperitoneally with C2 scFv (20 μg) 5 min before reperfusion. PBS served as the carrier for C2 scFv, while WT mice received an equal volume of PBS as the control group.^25^

### 2.4 Histological analysis

Liver tissues were immersed in a 10% formalin solution for histological analysis. Liver slices embedded in paraffin were stained with haematoxylin and eosin after undergoing dewaxing in xylene and rehydration using an ethanol gradient. Using a Leica microscope from Germany, hepatic histology was blindly assessed on a semi‐quantitative scale based on haematoxylin and eosin‐stained sections. Assessments of liver damage were conducted using the Suzuki score.^26^ Five random fields on each slide were assessed for necrotic area by loss of architecture, vacuolization, karyolysis and increased eosinophilia.

### 2.5 Levels of inflammatory cytokines in mouse serum

ELISA kits for IL‐6 (NeoBioscience Technology, Co., Ltd., China), IL‐1β (NeoBioscience Technology, Co., Ltd., China), TNF‐α (NeoBioscience Technology, Co., Ltd., China) and MCP‐1 (Ruixin Biotech, China) were used to determine levels of inflammatory cytokines in mouse serum. Mice serum was collected from sham‐ or IR‐operated mice 6 h post‐reperfusion. Serum levels of inflammatory factor were measured by ELISA assay according to the manufacturer's instructions.

### 2.6 Immunohistochemistry and TUNEL stainning

Immunohistochemical staining involved treating sections with 1% H_2_O_2_ for 20 min to eliminate endogenous peroxidase activity. Subsequently, a block with 5% bovine serum albumin (BSA) at room temperature for 1 h was followed by overnight incubation with primary antibodies at 4°C. Primary antibodies used were CD11b (Ab133357; 1:4000, Abcam), Ly6G (Ab238132; 1:2000, Abcam), IgM (Ab190369; 1:200, Abcam) and C3d (AF2655‐SP; 5 μg/mL, R&D). Secondary antibodies labelled with horseradish peroxidase (HRP) (RM3002 or RM3007; 1:500, Ray Antibody Biotech) were applied for 1 h at 37°C. Visualization was accomplished by utilizing 3,3‐diaminobenzidine (DAB) from Zhongshan Golden Bridge Biological Technology Co., Ltd. in Beijing, China, along with haematoxylin for counterstaining. The sections were examined under a Leica light microscope from Germany.

Assessment of cell death in liver tissue slices was done using TUNEL staining (11684817910, Roche) following the manufacturer's instructions. After dewaxing and hydrating the liver sections, protein digestion with protease K was carried out. The sections were then exposed to 3% H_2_O_2_‐methanol for 10 min, followed by incubation with TUNEL mixed reaction solution for 1 h at 37°C. The process was finished by staining with DAPI from Invitrogen in Carlsbad, CA, USA for 3 min at room temperature.

### 2.7 Statistical analysis

The data were processed and statistically analysed by GraphPad Prism version 5.0. Statistical significance among several groups was assessed through one‐way ANOVA, while significance between individual groups was determined using either a *t*‐test or nonparametric test. Data are expressed as the mean ± standard deviation (SD). *p* < 0*.05* indicated statistically significant differences.

## 3 RESULTS

### 3.1 Treatment with C2 scFv ameliorates liver injury following hepatic IR

**FIGURE 1:**
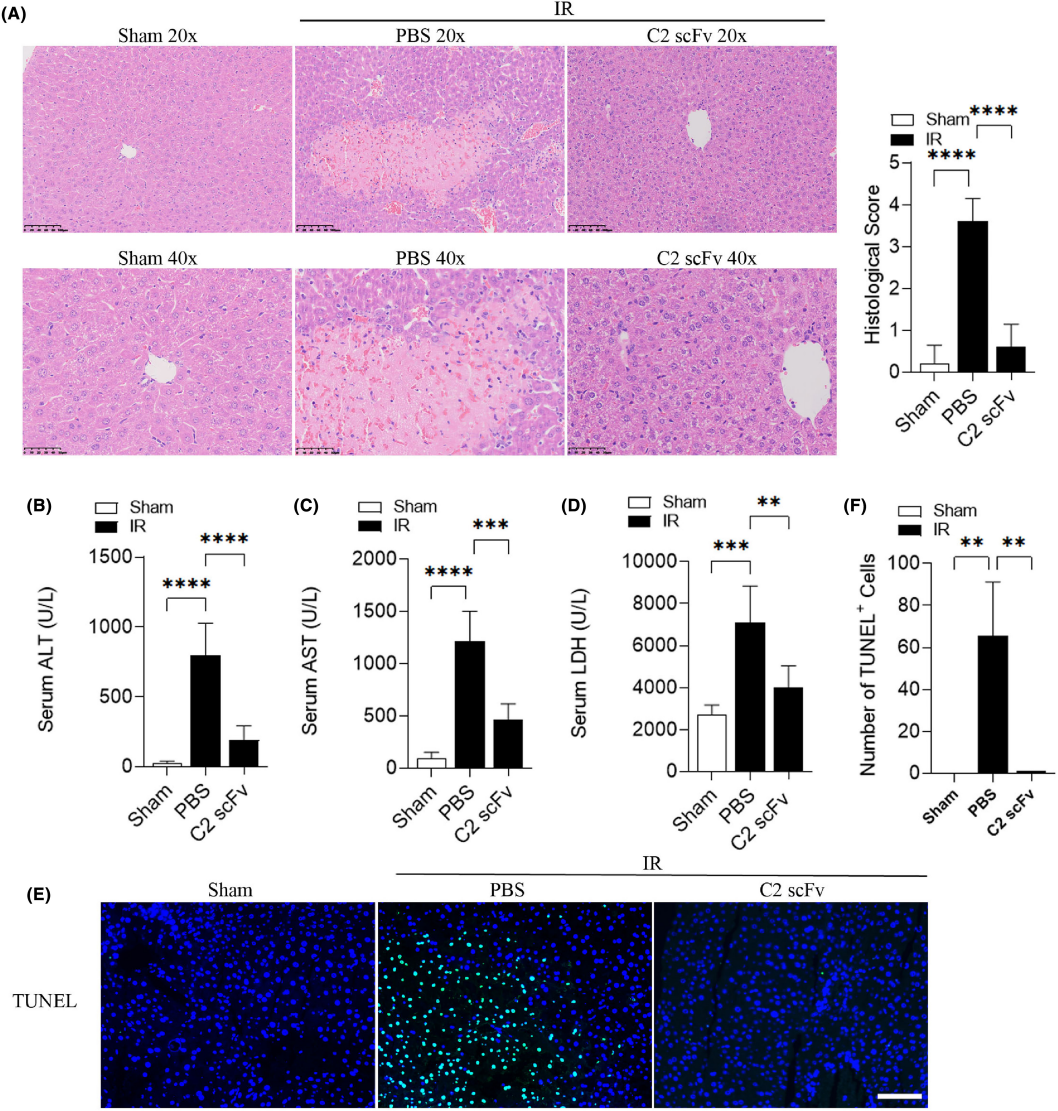
C2 scFv ameliorates hepatic IRI in WT mice. Representative haematoxylin and eosin staining and histological score (A), ALT (B), AST (C), LDH (D) (*n* = 5) and TUNEL staining (E) and quantification (F) (*n* = 3) in C2 scFv‐treated WT mice and their respective control mice after 30 min of hepatic ischemia followed by 6 h of reperfusion. (E) Scale bars represent 50 μm. Data are presented as the mean ± SD, ****p < 0.0001, ***p < 0.001, **p < 0.01.

In order to investigate the potential role of C2 scFv in hepatic IRI, we initially explored the therapeutic effect of C2 scFv on hepatic IRI in C57BL/6 mice. Haematoxylin and eosin examination of mouse liver sections showed that hepatocytes in Sham mice were tightly arranged and cord‐shaped, forming hepatic lobules (Figure 1A). The degree of liver damage in IR‐treated mice rapidly increased after reperfusion, manifested as inflammatory cell infiltration, liver cell hydration and necrosis, such as punctate necrosis, fragmented necrosis, strip necrosis and large sheet necrosis (Figure 1A). Compared with PBS‐treated IR mice, C2 scFv‐treated IR mice displayed mild liver damage after hepatic IR, with almost no necrotic areas and only a small amount of inflammatory cells (Figure 1A).

Additionally, levels of ALT, AST and LDH in the serum were significantly elevated in mice that underwent hepatic IR compared to Sham mice (Figure 1B–D). IR mice treated with C2 scFv showed notable decreases in serum levels of ALT, AST and LDH compared to that treated with PBS (Figure 1B–D). The number of TUNEL positive cells in the liver of mice that underwent hepatic IR was consistently higher compared to Sham mice. The liver of C2 scFv‐treated IR mice showed a significant decrease in the number of TUNEL positive cells compared to PBS‐treated IR mice (Figure 1E,F). Together, these findings indicate that C2 scFv has a potential therapeutic effect on hepatic IRI.

### 3.2 C2 scFv suppresses inflammation during hepatic IRI

**FIGURE 2:**
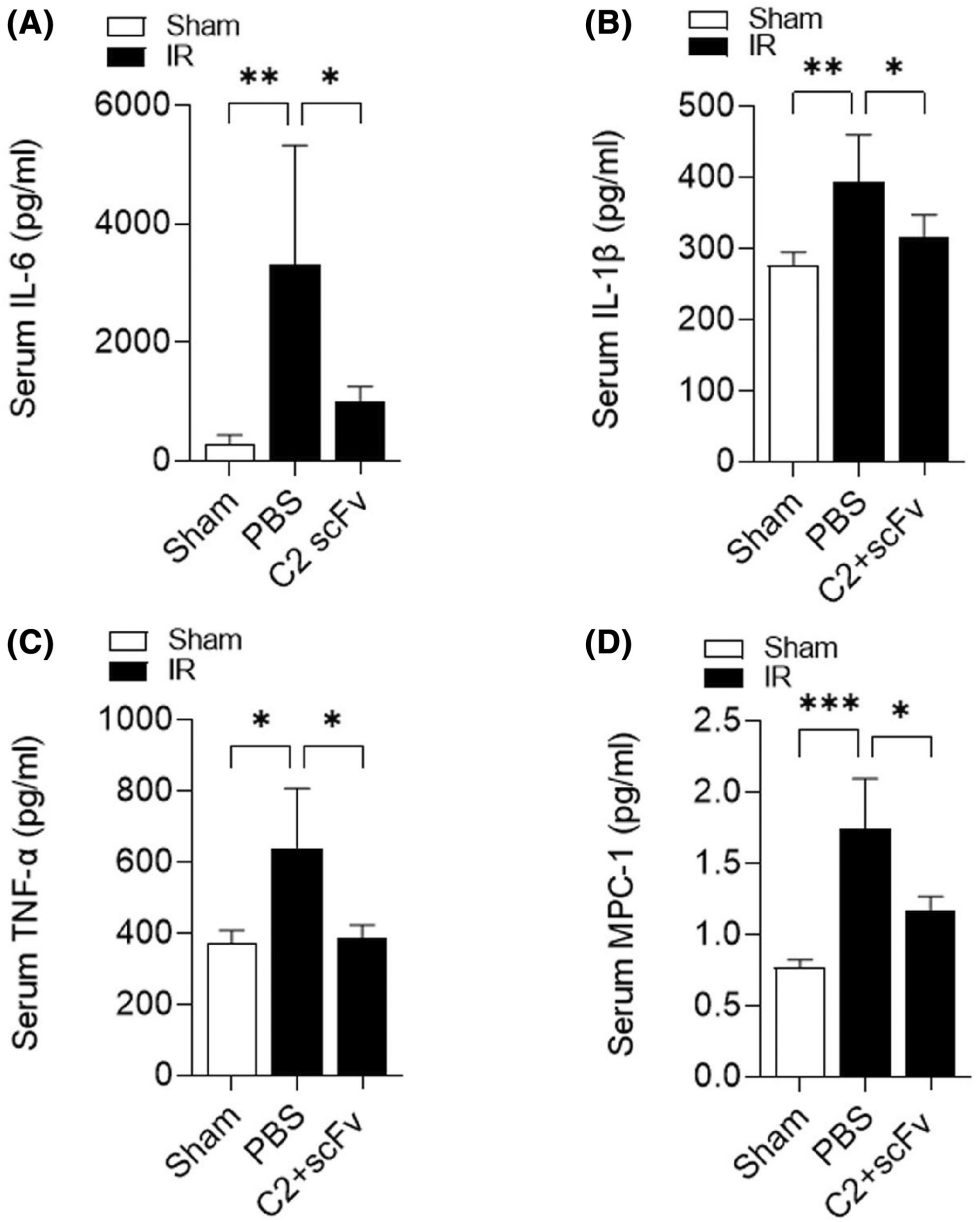
C2 scFv ameliorates inflammation during hepatic IRI. Serum IL‐6 (A), IL‐1β (B), TNF‐α (C) and MCP‐1 (D) levels in C2 scFv‐treated WT mice and their counterpart control mice subjected to 30 min of hepatic ischemia followed by 6 h of reperfusion. Data are presented as the mean ± SD. ***p < 0.001, **p < 0.01, *p < 0.05.

**FIGURE 3:**
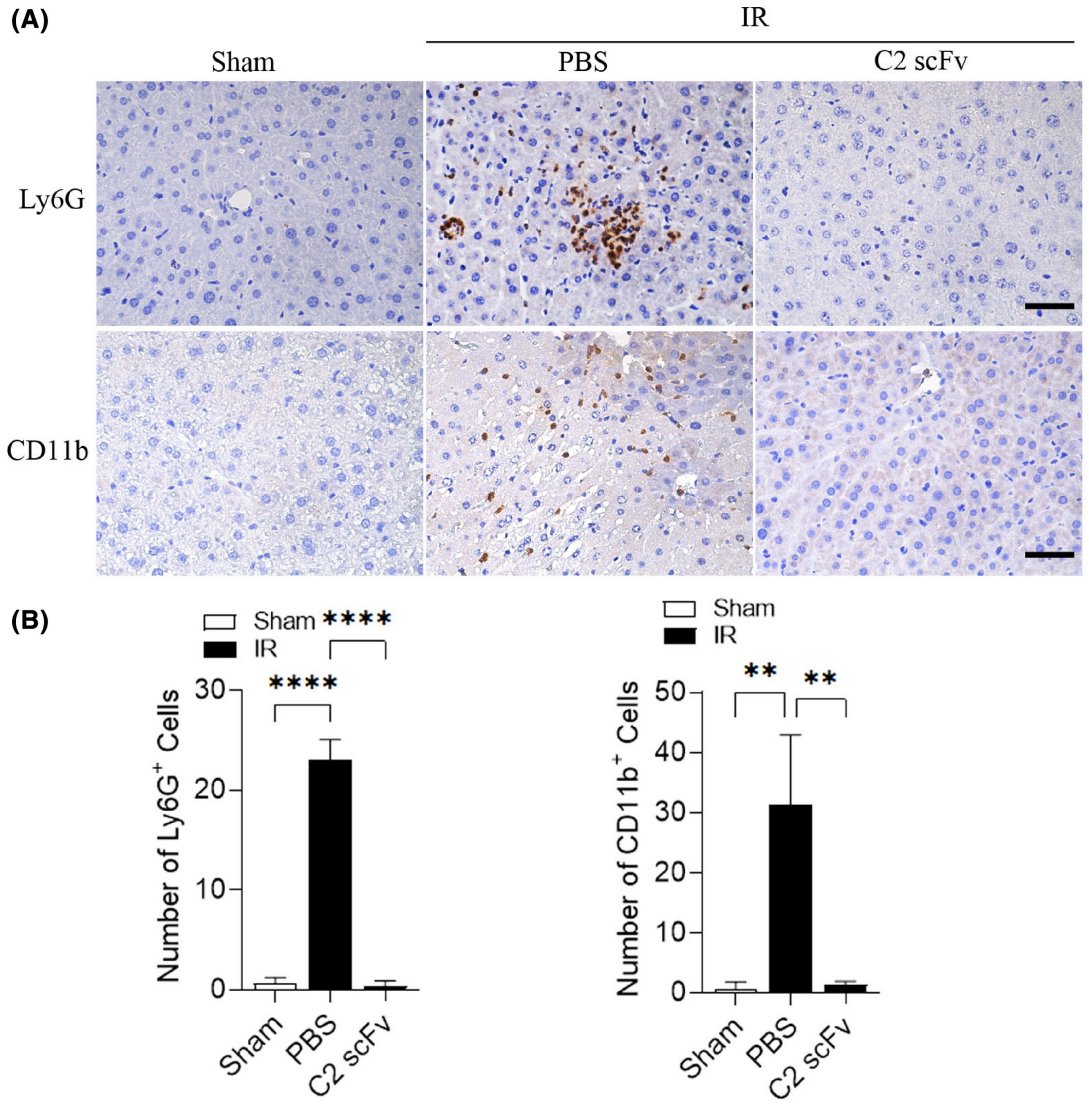
C2 scFv suppresses inflammatory cell infiltration during hepatic IRI. Representative immunohistochemical staining (A) and quantification (B) of infiltrating CD11b^+^, Ly6G^+^ cells in the livers of C2 scFv‐treated WT mice and their corresponding control mice following 30 min of hepatic ischemia followed by 6 h of reperfusion. Scale bars represent 50 μm. Data are presented as the mean ± SD, *n* = 3. ****p < 0.0001, **p < 0.01.

Due to the crucial function of the inflammatory response in hepatic IRI^26^ and the potential therapeutic effect of C2 scFv on hepatic IRI, we investigated the relationship between C2 scFv treatment and inflammation during hepatic IRI. Serum levels of inflammatory cytokines (IL‐6, TNF‐α and IL‐1β) and MCP‐1 were significantly increased in PBS‐treated mice after hepatic IRI (Figure 2A–D). Consistent with the results of above immunohistochemistry experiments, IL‐6, IL‐1β, TNF‐α and MCP‐1 levels were markedly lower in C2 scFv‐treated IR mice than in PBS‐treated IR mice. Moreover, immunohistochemical staining revealed that a notable rise in the quantity of Ly6G^+^ and CD11b^+^ cells in the livers of mice that underwent hepatic IR in comparison to Sham mice. Moreover, the numbers of inflammatory cells after hepatic IRI dramatically decreased in the liver of C2 scFv‐treated mice compared with that in PBS‐treated mice (Figure 3A,B). Collectively, these data demonstrate that C2 scFv suppresses inflammation during hepatic IRI.

### 3.3 C2 scFv reconstitutes hepatic IRI in Rag1^−/−^ mice

**FIGURE 4:**
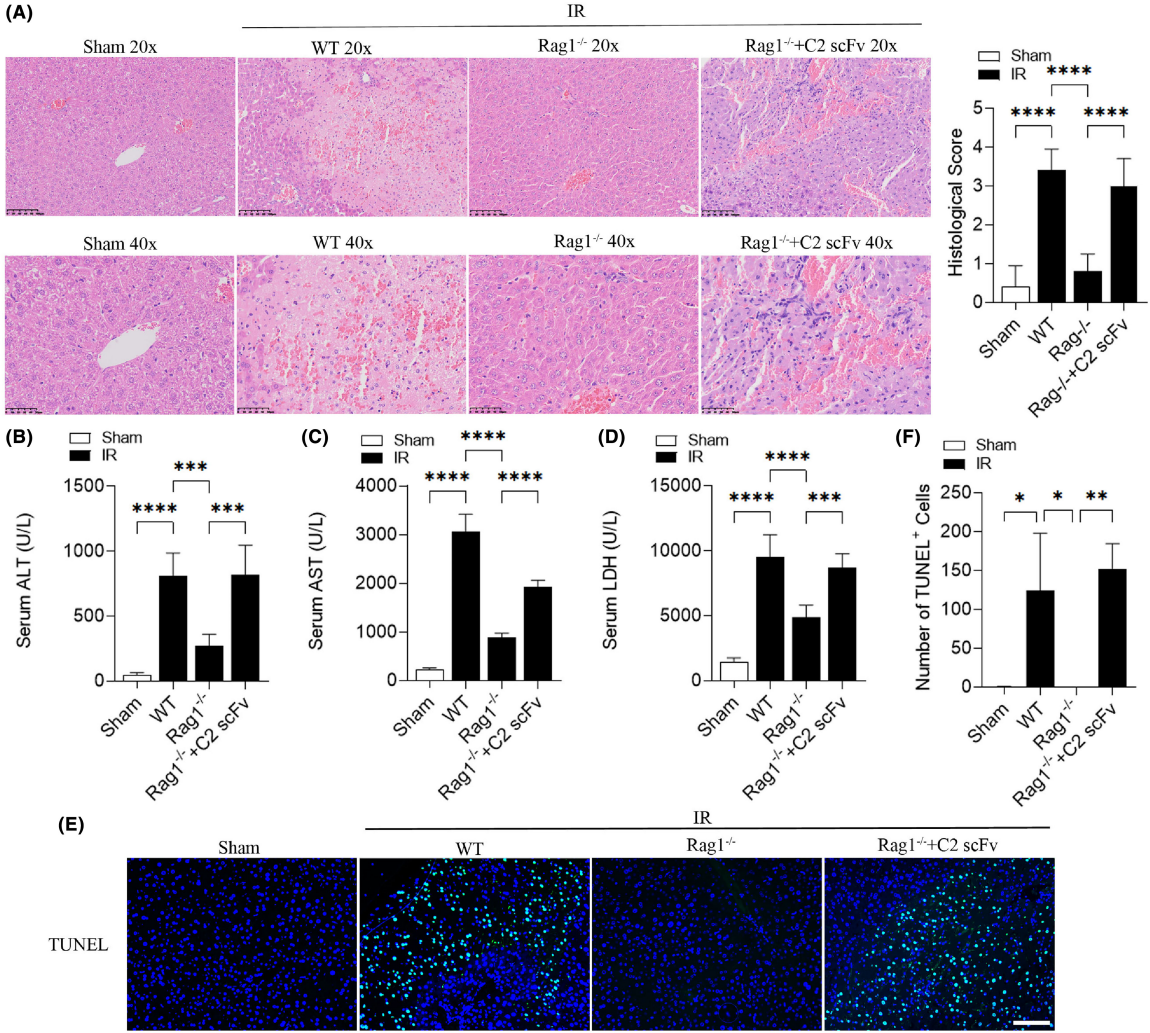
C2 scFv reconstitutes hepatic IRI in Rag1^−/−^ mice. Illustrative haematoxylin and eosin staining and histological score (A), ALT (B), AST (C), LDH (D) (*n* = 5) and TUNEL staining (E) and quantification (F) (*n* = 3) in C2 scFv‐treated Rag1^−/−^ mice and their corresponding control mice subjected to 30 min of hepatic ischemia followed by 6 h of reperfusion. (E) Scale bars represent 50 μm. Data are presented as the mean ± SD. ****p < 0.0001, ***p < 0.001, **p < 0.01, *p < 0.05.

Marshall and colleagues^25^ have shown that B4 mAb and C2 mAb can reconstitute the hepatic IRI in Rag1^−/−^ mice, so we researched the potential role of C2 scFv in reconstituting hepatic IRI in Rag1^−/−^ mice. In comparison to WT mice, Rag1^−/−^ mice appeared a protective effect against hepatic IRI, as indicated by a significantly lower histological score after 30 min ischemia and 6 h reperfusion (Figure 4A). The liver lobule structure could be clearly seen in Rag1^−/−^ mice, and the liver cells were arranged radially around the central vein and the liver lobule boundary was very obvious. Moreover, C2 scFv‐treated IR Rag1^−/−^ mice exhibited exacerbated liver injury, characterized by increased necrotic area and higher histological score, than in corresponding controls (Figure 4A). Similarly, serum ALT, AST and LDH levels were statistically higher in WT IR mice than in WT Sham mice, while those in Rag1^−/−^ mice underwent hepatic IR were significantly decreased (Figure 4B–D). In contrast, serum ALT, AST and LDH levels were significantly increased in C2 scFv‐treated Rag1^−/−^ mice underwent hepatic IR (Figure 4B–D). In addition, the number of TUNEL positive cells showed a similar change pattern (Figure 4E,F). In summary, these results suggest that C2 scFv reconstitutes the hepatic IRI in Rag1^−/−^ mice.

### 3.4 C2 scFv exacerbates inflammation following reconstitution of hepatic IRI in Rag1^−/−^ mice

**FIGURE 5:**
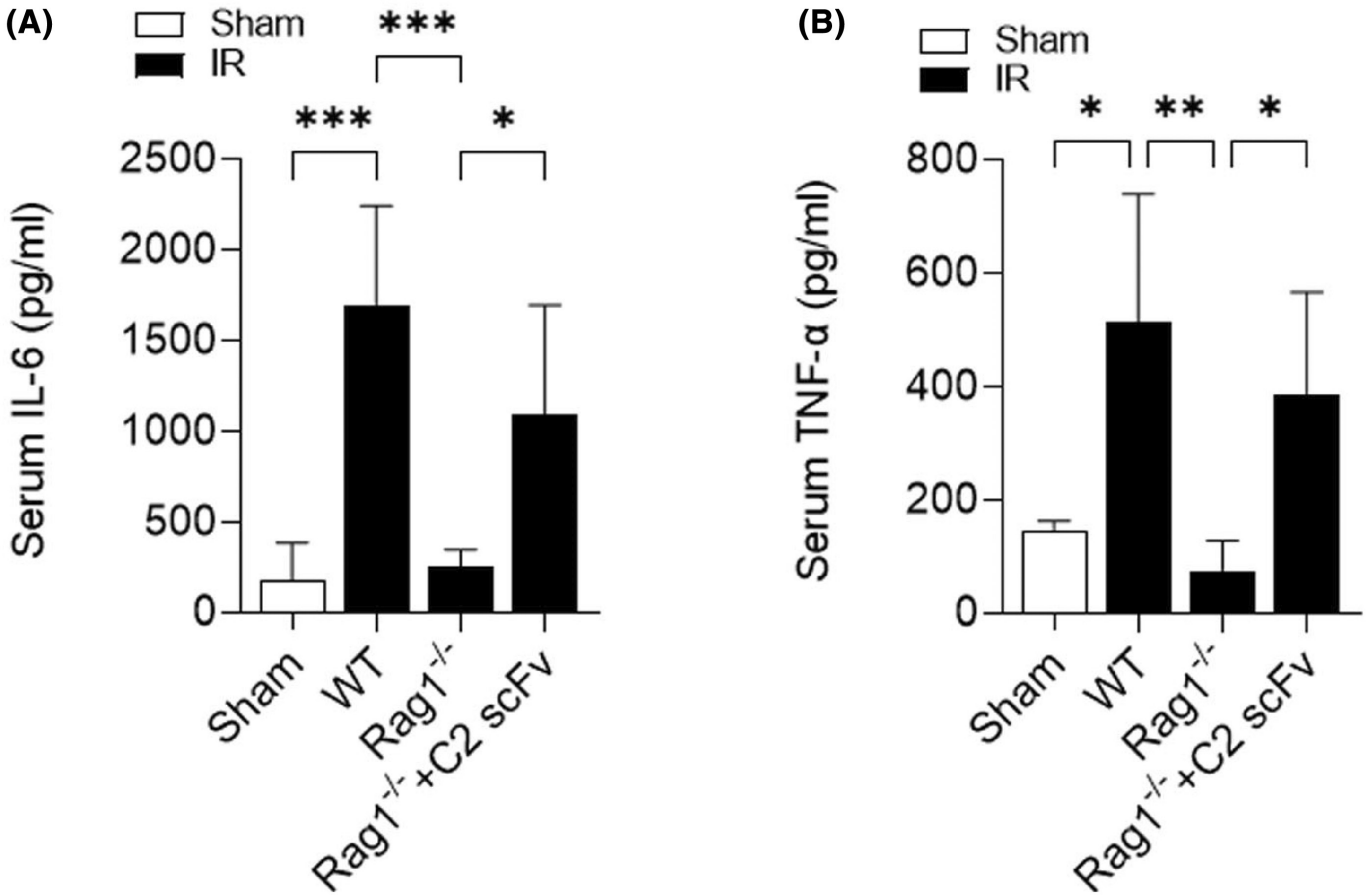
C2 scFv exacerbates inflammation following reconstitution of hepatic IRI in Rag1^−/−^ mice. Serum IL‐6 (A) and TNF‐α (B) levels in C2 scFv‐treated Rag1^−/−^ mice and their corresponding control mice following 30 min of hepatic ischemia followed by 6 h of reperfusion. Data are presented as the mean ± SD. ***p < 0.001, **p < 0.01, *p < 0.05.

**FIGURE 6:**
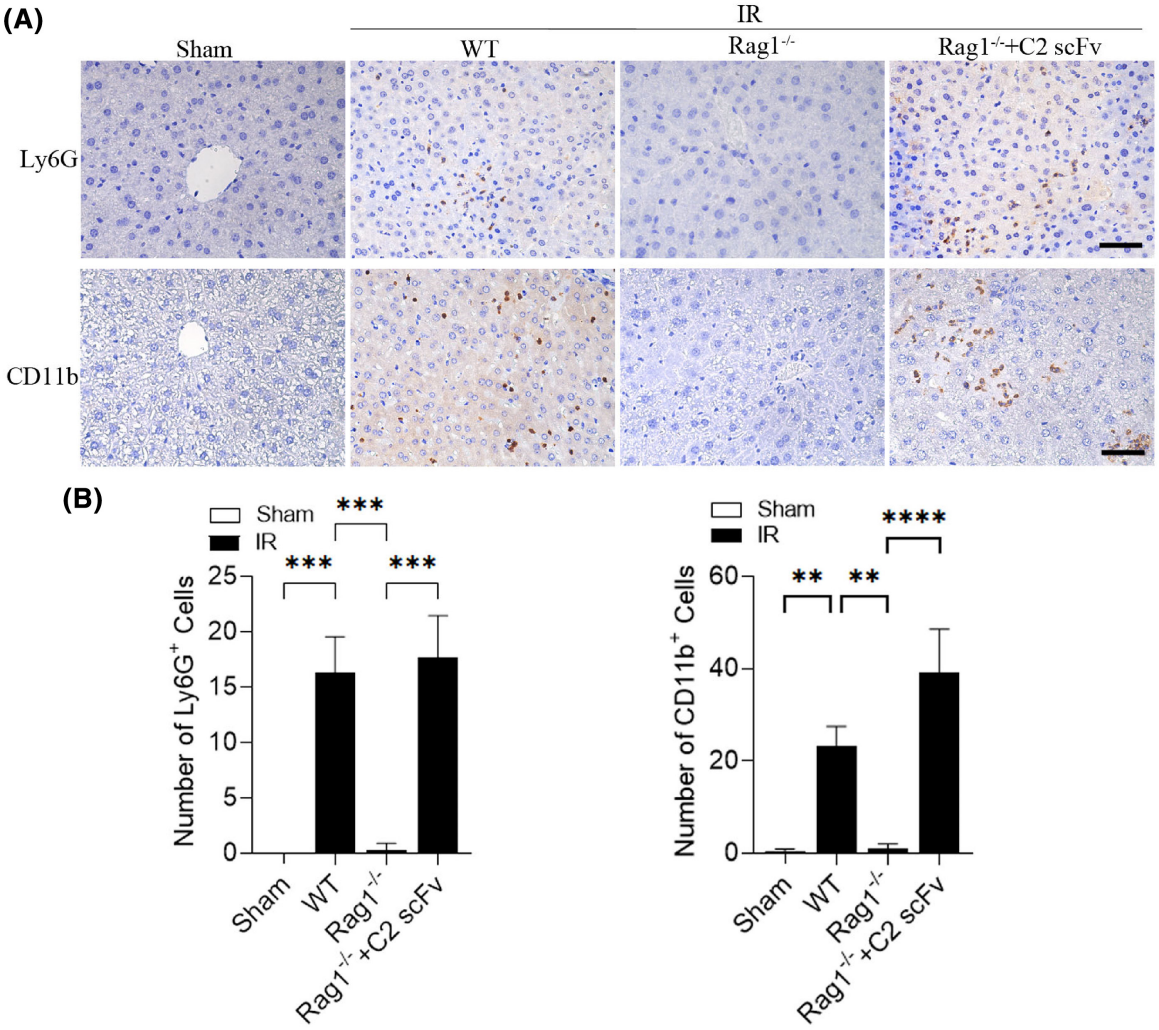
C2 scFv enhances inflammatory cell infiltration during hepatic IRI in Rag1^−/−^ mice. Analysis of infiltrating CD11b^+^ and Ly6G^+^ cells in the livers of C2 scFv‐treated Rag1^−/−^ mice and their corresponding control mice after 30 min of hepatic ischemia followed by 6 h of reperfusion, shown through representative immunohistochemical staining (A) and quantification (B). Scale bars represent 50 μm. Data are presented as the mean ± SD, *n* = 3. ****p < 0.0001, ***p < 0.001, **p < 0.01.

The above results showed that C2 scFv could inhibit inflammation during hepatic IRI in WT C57BL/6 mice. Following this, we examined how C2 scFv contributes to the inflammatory reaction following hepatic IRI reconstitution in Rag1^−/−^ mice. Levels of IL‐6 and TNF‐α in the serum showed a significant increase in WT mice following hepatic IRI, whereas they were significantly reduced in Rag1^−/−^ mice that underwent hepatic IR (Figure 5A,B). Furthermore, C2 scFv‐treated Rag1^−/−^ mice subjected to hepatic IR exhibited significantly elevated IL‐6 and TNF‐α levels (Figure 5A,B). Immunohistochemical examination showed a notable increase in Ly6G^+^ and CD11b^+^ cells in WT IR mice compared to WT Sham mice, with a significant decrease in Rag1^−/−^ mice subjected to hepatic IR (6A‐6B). Moreover, the presence of inflammatory cells in the liver following hepatic IRI was notably higher in the C2 scFv‐treated Rag1^−/−^ mice than in the controls (Figure 6A,B). Taken together, these observations indicate that C2 scFv induces inflammation in Rag1^−/−^ mice during hepatic IRI.

### 3.5 IgM mAb and C2 scFv localize to injured tissue and play crucial roles in complement activation

**FIGURE 7:**
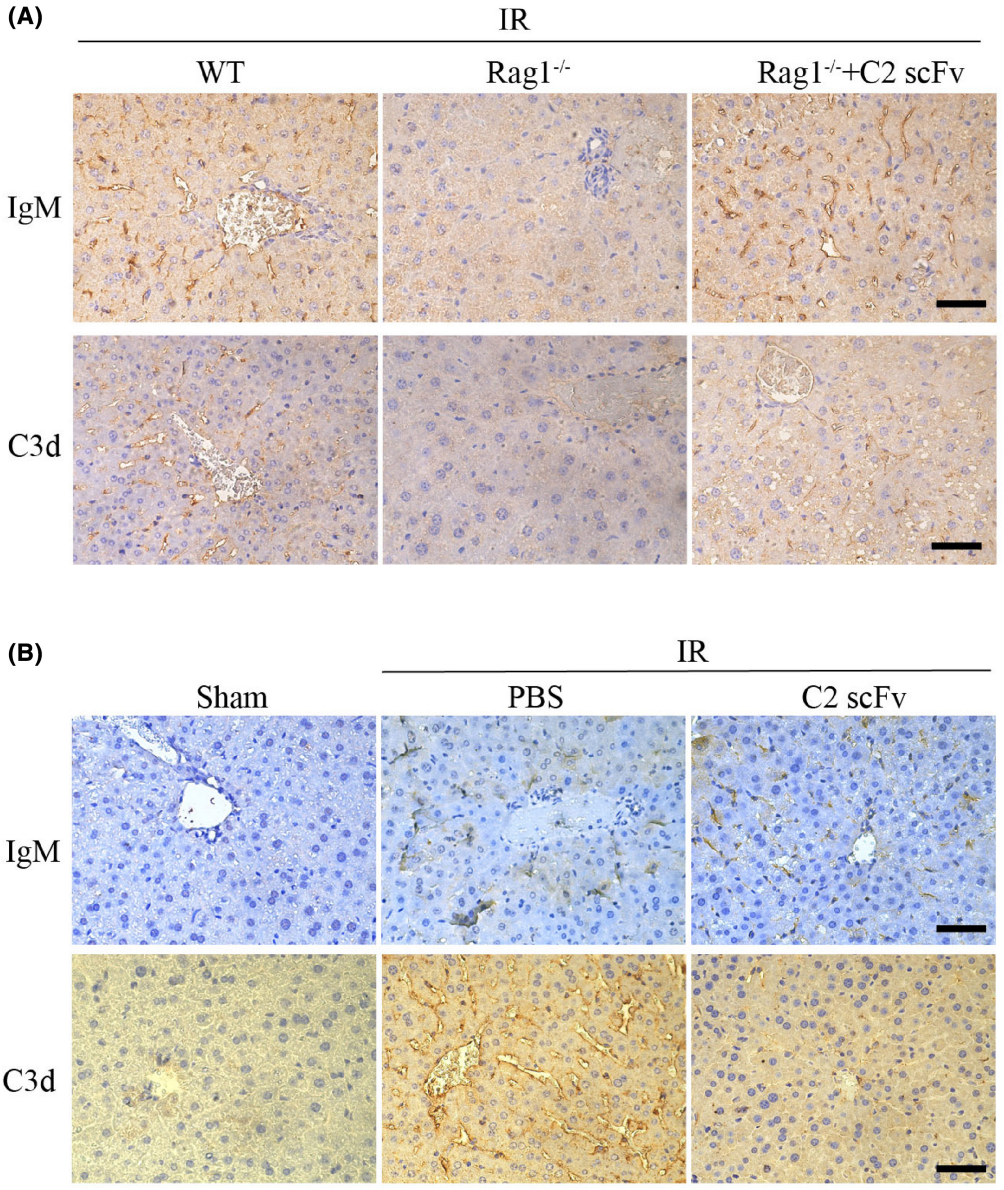
The role of IgM in complement activation. (A) Restoration of IgM and C3d deposition in Rag1^−/−^ mice treated with C2 scFv following hepatic IRI. Immunohistochemical staining for IgM and C3d was performed in mice subjecting 30 min hepatic ischemia followed by 6 h reperfusion. (B) Reduction in C3d deposition in WT mice treated with C2 scFv following hepatic IRI. Immunohistochemical staining for IgM and C3d in C2 scFv‐treated WT mice and their respective control mice after 30 min of hepatic ischemia followed by 6 h of reperfusion. Scale bars represent 50 μm.

Some studies have reported the presence of IgM deposition during hepatic IRI. To explore the correlation between IgM deposition and complement activation, we analysed IgM and complement activation, indicated by C3d deposition, in necrotic regions of the liver at 6 h post‐reperfusion. High levels of IgM and C3d deposition were observed in post‐ischemic liver sections while much weaker IgM and C3d expression were detected in post‐ischemic liver sections from Rag1^−/−^ mice (Figure 7A). Conversely, post‐ischemic liver sections from Rag1^−/−^ mice reconstituted with C2 scFv showed high levels of deposited IgM and C3d following hepatic IR (Figure 7A). Collectively, these findings suggest that C2 scFv can activate complement in Rag1^−/−^ mice during hepatic IRI. Moreover, high levels of IgM deposition were observed in C57BL/6 IR mice treated with PBS and C2 scFv (Figure 7B). However, the expression levels of C3d were significantly reduced in C2 scFv‐treated IR mice compared to those treated with PBS (Figure 7B), suggesting that excessive exogenous of C2 scFv can inhibit complement activation. Thus, the novel epitope of phospholipid subsets recognized by C2 scFv may provide a therapeutic target for inhibiting hepatic IRI.

## 4 DISCUSSION

It is essential to note that complement activation products play a crucial role in the development of hepatic IRI.^27^ Components of the complement system have been proven to exacerbate liver damage while to promote liver regeneration during hepatic IRI.^28^, ^29^ IgM can activate complement through the classical pathway.^30^ The combination of the two tail ends of the pentamer IgM can also activate the complement bypass.^31^ The more complement proteins are activated, the more immune cells are attracted to the pathogen. Excess complement proteins and immune cells cause inflammation and tissue damage. Studies demonstrated that IgM binding to injured tissue precedes complement activation during skeletal muscle ischemia reperfusion.^32^ Furthermore, a recent study reported that IgM antibodies against acetylated proteins may be the starting point of the anti‐modified protein antibody response in rheumatoid arthritis.^33^ Here, we demonstrate that C2 scFv, a new single‐chain antibody construct derived from IgM, is vital for triggering complement activation during hepatic IRI.

In this study, we have demonstrated that C2 scFv plays an important role in the improvement of hepatic IRI. First, to detect the therapeutic effect of C2 scFv, C57BL/6 mice were intraperitoneally injected with C2 scFv 5 min before reperfusion. We found that C2 scFv effectively blocked IgM binding in C57BL/6 mice with a complete natural antibody profile, providing protection against hepatic IRI. Compared with PBS‐treated mice, C2 scFv‐treated mice displayed mild liver damage after hepatic IR, with almost no necrotic areas and only a small amount of hydration liver cell and inflammatory cells. Additionally, C2 scFv‐treated mice displayed significant reduction in serum ALT, AST and LDH levels. Similarly, the number of TUNEL positive cells in C2 scFv‐treated mice was significantly reduced. Moreover, C2 scFv‐treated mice exhibited a notable reduction in inflammatory cells after hepatic IRI than PBS‐treated mice. The serum IL‐6, IL‐1β and TNF‐α, and MPC‐1 levels were also severely suppressed by C2 scFv. In summary, C2 scFv can reduce hepatic IRI by inhibiting inflammation and liver cell death.

In order to further investigate whether C2 scFv protects against hepatic IRI by affecting complement activation, we reconstituted liver IRI in Rag1^−/−^ mice. Rag1^−/−^ mice were intraperitoneally injected with 20 μg of C2 scFv 5 min before reperfusion. Indeed, The protective effect of Rag1−/− mice against hepatic IRI was reversed by C2 scFv treatment. Compared with corresponding control mice, C2 scFv‐treated mice exhibited more severe liver injury after hepatic IR, as evidenced by increased necrotic areas, elevated serum levels of ALT, AST and LDH, and an increased number of TUNEL positive cells. Moreover, the numbers of inflammatory cells and serum IL‐6 and TNF‐α levels were remarkably higher in C2 scFv‐treated mice than in corresponding controls after hepatic IRI. Similarly, B4 IgM was capable of inducing hepatic IRI in Rag1^−/−^ mice.^25^ However, our results imply that even a single‐chain antibody derived from C2 IgM can also reconstitute hepatic IRI in Rag1^−/−^ mice.

It seems controversial that C2 scFv can both reduce hepatic IRI in WT mice and reconstitute hepatic IRI in Rag1^−/−^ mice. K. Marshall and colleagues proposed that DAMPs targeted by IgM and by other effector molecules of the innate immune system, such as phospholipids and apoptotic cells, activate complement synergistically.^25^ Moreover, this inflammatory cascade does not advance in a linear fashion; rather, it exponentially amplifies as all of these elements come into play. Blocking any of these components in the early stages can significantly hinder the cascade and subsequent damage.^25^ Our results were in agreement with the hypothesis. Deficient of IgM in Rag1^−/−^ mice resulted in tolerance to hepatic IRI, suggesting IgM is essential to the progression of hepatic IRI. We found that IgM and C3d deposition were significantly decreased in Rag1^−/−^ mice underwent hepatic IR while C2 scFv reconstituted IgM and C3d deposition, suggesting that even supplement with a single‐chain IgM antibody construct is able to restore complement activation in Rag1^−/−^ mice underwent hepatic IR. Therefore, in immune deficiency situation, C2 scFv can replace IgM to restore complement activation in hepatic IR. This is consistent with the idea that natural IgM antibodies are important components of the immune system. When detecting the levels of C3d deposition in post‐ischemic liver sections from C57BL/6 mice, we found that the expression levels of C3d were significantly reduced in C2 scFv‐treated mice compared to those treated with PBS. These results suggested that C2 scFv can reduce C3d deposition in WT mice with hepatic IRI. IgM can recognize post‐ischemic neoepitopes following reperfusion of tissues and activate complement. DAMPs are targeted by IgM and other effector molecules of the innate immune system, such as phospholipids and apoptotic cells, and synergistically activate complement.^25^ Excessive exogenous supplement of C2 scFv occupied all epitopes of phospholipid subsets which could otherwise bound to other effector molecules of the innate immune system, and thus blocked complement activation and subsequently inhibited hepatic IRI in WT mice. C2 scFv exhibiting both protective and exacerbating effects on hepatic IRI in different contexts, additional investigation to fully elucidate the underlying mechanisms and potential implications for clinical translation are needed.

Taken together, our study suggests that C2 scFv ameliorates hepatic IRI by inhibiting complement activation and administration of C2 scFv may serve as a potential therapy for hepatic IRI.

## AUTHOR CONTRIBUTIONS

**Zhi Yang:** Conceptualization (lead); data curation (lead); methodology (lead); validation (lead); writing – original draft (lead). **Junfei Jin:** Conceptualization (lead); formal analysis (lead); funding acquisition (lead); methodology (lead); project administration (lead); resources (lead); supervision (lead); writing – review and editing (lead). **Chunmei Li:** Conceptualization (equal); data curation (equal); formal analysis (equal); investigation (equal); methodology (equal); validation (equal). **Yongqin Wang:** Conceptualization (supporting); data curation (supporting); methodology (supporting); project administration (supporting); supervision (supporting). **Wei Dong:** Conceptualization (supporting); formal analysis (supporting); methodology (supporting). **Moujie Yang:** Conceptualization (supporting); investigation (supporting); validation (supporting).

## CONFLICT OF INTEREST STATEMENT

The authors involved in this study have disclosed no conflicts of interest pertaining to this manuscript.

## Supporting information


Figure S1.

## Data Availability

No publicly available data or shared data are cited. All data needed to evaluate the conclusion of the current study are present in the paper.
