# Supplementary material for: A single‐chain antibody construct with specificity of a natural IgM antibody reduces hepatic ischemia reperfusion injury in mice
Source: J Cell Mol Med. 2024 Apr 10;28(8):e18291. doi: 10.1111/jcmm.18291 (PMC11005456; doi:10.1111/jcmm.18291)
Supplement: Supplementary file 1 — Figure S1. [file JCMM-28-e18291-s001.docx]

**A** **single-chain antibody construct with specificity of a natural IgM antibody reduces hepatic ischemia reperfusion injury in mice**

Zhi Yang^1,2,3,4,#^, Chunmei Li^1,2,3,4,#^, Yongqin Wang^1^, Wei Dong^1^, Moujie Yang^1,2^, Junfei Jin^1,2,3,4*^

^1^Guangxi Key Laboratory of Molecular Medicine in Liver Injury and Repair, the Affiliated Hospital of Guilin Medical University, Guilin, 541001, Guangxi, China

^2^Guangxi Health Commission Key Laboratory of Basic Research in Sphingolipid Metabolism Related Diseases, the Affiliated Hospital of Guilin Medical University, Guilin, 541001, Guangxi, China

^3^China-USA Lipids in Health and Disease Research Center, Guilin Medical University, Guilin, 541001, Guangxi, China

^4^Laboratory of Hepatobiliary and Pancreatic Surgery, the Affiliated Hospital of Guilin Medical University, Guilin, 541001, Guangxi, China

*Correspondence: Junfei Jin (junfeijin@glmc.edu.cn), Guangxi Key Laboratory of Molecular Medicine in Liver Injury and Repair, the Affiliated Hospital of Guilin Medical University, Guilin, 541001, Guangxi, China; Tel.: +86 773 2862270; Fax: +86 773 2810411, Address: 15 Lequn Road, Guilin, 541001, Guangxi, China.

**Supporting Figures**


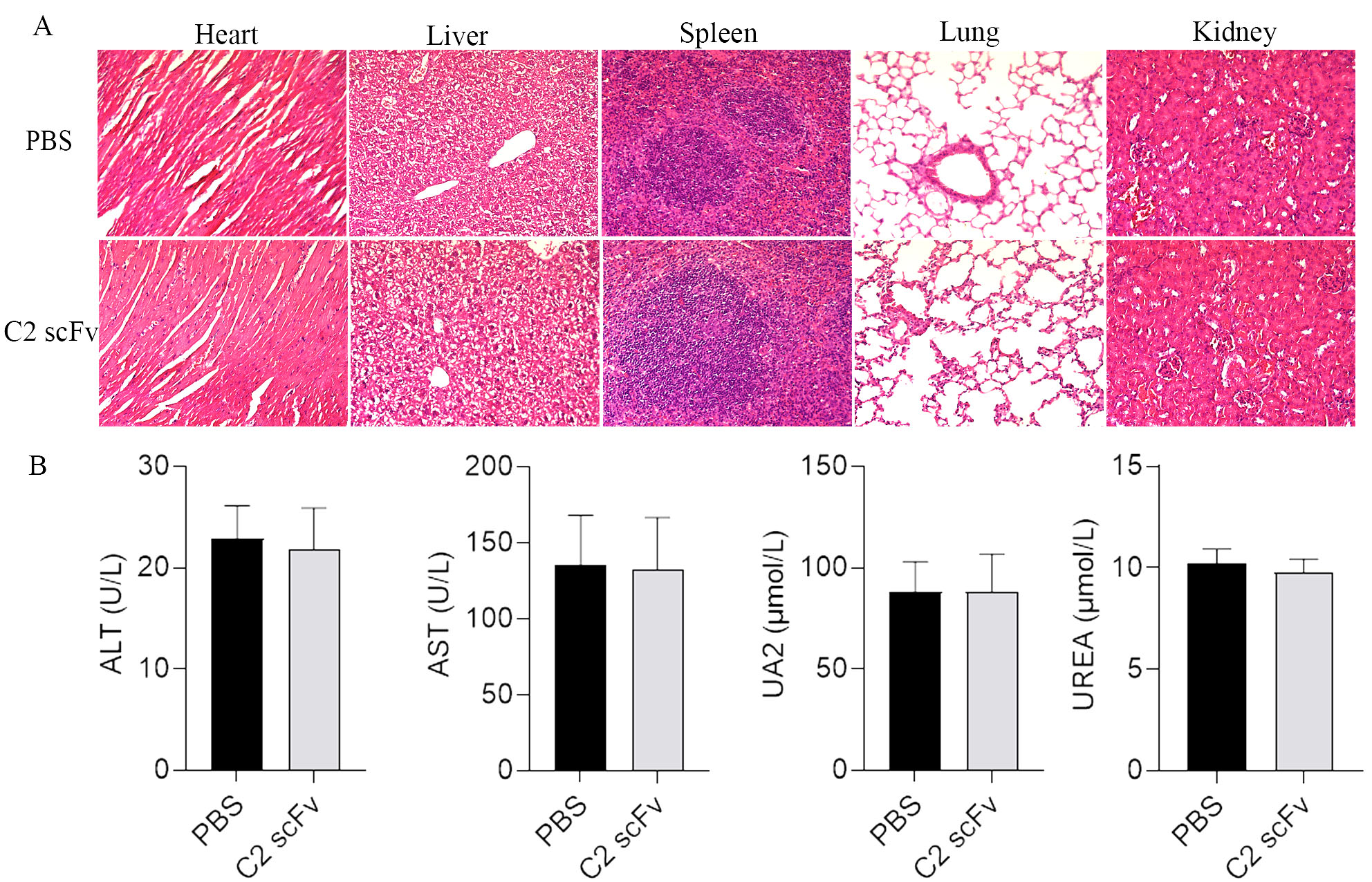


**Fig. S1 Safety evaluation of C2 scFv treatment for 24 hours**

**(A)** H&E staining images of heart, liver, spleen, lung, and kidney. **(B)** ALT, AST, UA2 and UREA analysis. Data were presented as the mean±SD. n=5.
